# Pixel-Label-Based Segmentation of Cross-Sectional Brain MRI Using Simplified SegNet Architecture-Based CNN

**DOI:** 10.1155/2018/3640705

**Published:** 2018-10-28

**Authors:** Bijen Khagi, Goo-Rak Kwon

**Affiliations:** Department of Information and Communication Engineering, Chosun University, 375 Seosuk-Dong, Dong-Gu, Gwangju 501-759, Republic of Korea

## Abstract

Using deep neural networks for segmenting an MRI image of heterogeneously distributed pixels into a specific class assigning a label to each pixel is the concept of the proposed approach. This approach facilitates the application of the segmentation process on a preprocessed MRI image, with a trained network to be utilized for other test images. As labels are considered expensive assets in supervised training, fewer training images and training labels are used to obtain optimal accuracy. To validate the performance of the proposed approach, an experiment is conducted on other test images (available in the same database) that are not part of the training; the obtained result is of good visual quality in terms of segmentation and quite similar to the ground truth image. The average computed Dice similarity index for the test images is approximately 0.8, whereas the Jaccard similarity measure is approximately 0.6, which is better compared to other methods. This implies that the proposed method can be used to obtain reference images almost similar to the segmented ground truth images.

## 1. Introduction

Deep neural networks have been highly successful in segmenting outdoor scenes with high complexity, dissimilar patterns, variable texture, and wide pixel range. In the present study, this model is used for segmenting MRI images of the brain, which are relatively simpler than outdoor scenes. The precise segmentation of a 2D image has always been a challenging task, and various approaches have been proposed for better accuracy, such as supervised and unsupervised, manual and automatic, and standalone and neural network-based techniques. Similarly, deep convolutional neural networks (CNNs) have been effective in machine learning and have had impact on various industrial, medical, and commercial fields. Generally, image segmentation is the process of presenting and partitioning image content into distinguishable parts. Moreover, segmentation methods from edge detection as well as supervised and unsupervised methods have been proposed. Similarly, neural networks have been developed for medical image processing, particularly in MRI image segmentation and Alzheimer's disease classification [1, 2]. Brain MRI segmentation is fundamental in several clinical applications and influences the outcome of the entire analysis because various processing operations rely on accurate segmentation of anatomical and structural regions. For instance, MRI segmentation is frequently used for calculating and imagining different brain structures, delineating lesions, analyzing brain development, and image-guided intrusions and surgical preparation. In MRI, tissue is heterogeneously concentrated in terms of intensity owing to the bias field and the partial volume effect that reflects the actual content of the brain, namely, white matter (WM), gray matter (GM), and cerebrospinal fluid (CSF). Therefore, accurate and selective methods should be chosen.

In contrary to existing methods [3], which have a certain way of feature extraction and criteria like thresholding, contours, and clustering, this method has been used extensively by many researchers and found to be excellent in case of MRI segmentation as well. But on the contrary, deep neural network are now proving to be better, highly computational for large data, and powerful because of encoder-decoder-based network or CNN architecture. The features are automatically investigated from low level features like edge, blob, and line to high level features like color, shape, and detail in a hierarchical manner by each layer. The activation layer like ReLu helps to make those features more clear and computable. Hence, we can easily get our segmentation result using our model. The only problem will be to train the network as it requires a large amount of ground truth and design the network appropriately.

## 2. Background and Methodology

### 2.1. Semantic Segmentation

In semantic segmentation, the image is segmented on a pixel-label basis, that is, each pixel is associated with a certain defined class. Its applications include scene understanding, autonomous driving, object recognition, machine translation, and machine vision. Semantic segmentation has been improved by using full CNNs [4] and deep CNNs [5–8]. These neural networks are trained in an end-to-end, pixel-to-pixel manner on each layer for image segmentation.

### 2.2. SegNet Layer

The SegNet layer is a deep full CNN architecture adapted for semantic segmentation that was proposed by Vijay Badrinarayanan et al. [5]. Generally, the semantic segmentation approach is used for outdoor, indoor, and road scenes mostly for a large number of classes. SegNet was originally designed for scene understanding applications. Hence, it should be efficient in terms of memory, operation, and computational time. It is also considerably be smaller in terms of the number of trainable parameters than other competing architectures, and it can be used in training end-to-end pixel-label classes using stochastic gradient descent and the cross-entropy loss function.

The encoder used in SegNet is identical to the convolutional layers in VGG16 [9]. The fully connected layers of VGG16 have been removed in SegNet, and thus the encoder network is considerably reduced and easier to train compared to other recent architectures [5, 6, 10, 11]. The most important constituent of SegNet is the encoder-decoder network, which consists of a hierarchy of downsampling encoders matching each upsampling decoder with associated feature vectors cycling inside them.

### 2.3. CNN and Architecture

CNNs have always been important in machine learning; by using various types of neural networks, systematic training and testing of image and pixel labels can be performed. The encoder network used here consists of convolution layers of 64 filters, each of size 3 × 3, manually padded, followed by batch normalization and ReLu activation unit and repeatedly followed by same convolution, batch normalization, and ReLu for proper downsampling and robust feature extraction. Same is the case with decoder convolution network but firstly unpool layer and then convolution layer following batch normalization and ReLu.

**Figure 1:**
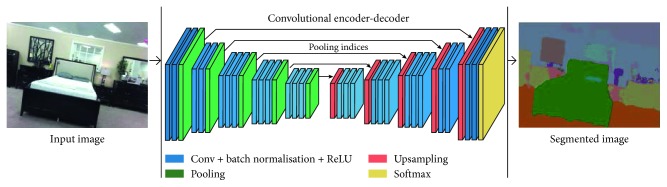
SegNet architecture pictorial representation as presented by Vijay Badrinarayanan et al. [5].

**Figure 2:**
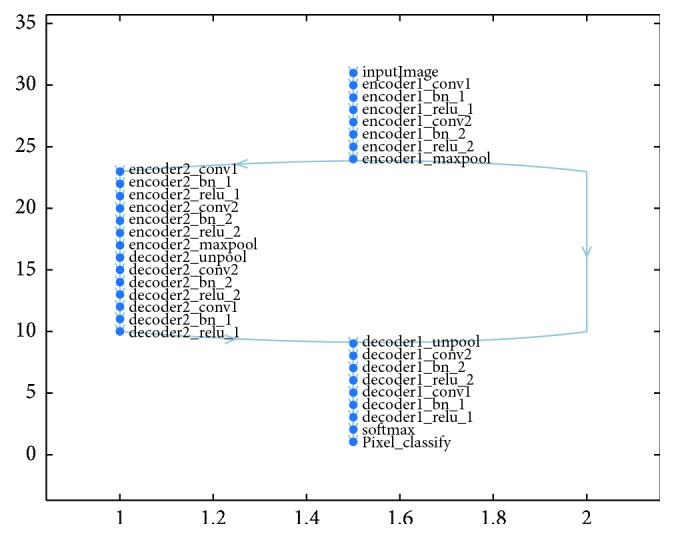
Schematic representation of 31 layers and 34 connections used in proposed CNN Network.

Proposed CNN has an encoder network and a matching decoder network, which is followed by a final pixel-based classification layer. This architecture is shown in Figure 1. To simplify the architecture, two encoder and two decoder networks have been employed: *encoder1* is mapped to *decoder1*, and *encoder2* is mapped to *decoder2*. *encoder1* consists of *encoder1_conv1*, *encoder1_bn_1*, *encoder1_relu_1*, and *encoder1_maxpool_1* in hierarchical order, whereas *dencoder1* consists of *dencoder1_unpool_1*, *dencoder1_conv1*, *dencoder1_bn_1*, and *dencoder1_relu_1*. *encoder2* and *decoder2* are similarly structured. Here, *encoder1* is followed by *encoder2*, and *dencoder2* is followed by *dencoder1*, as shown in Figure 2. The first 13 layers constitute an encoder network that performs the convolution with 64 filter banks of size 3 × 3 to obtain sets of features along with batch normalization in a minibatch set of 8 images. ReLU acts as an activation function *f*(*x*) = max (0, *x*), which can be used by neurons, as any other activation function, to eliminate negative values. Thereafter, the max pooling layer with a 2 × 2 window and stride size 2 (nonoverlapping window) is executed, so that the resulting output is downsampled by a factor of 2. Multiple layers of max pooling downsampling are used to achieve more translation invariance and robust pixel classification. Similarly, the decoder in the decoder network upsamples the input layer feature maps unpooling the memorized max pooling indices with the location of maximum feature values from the corresponding encoder feature maps. It is followed by the convolution and batch normalization layers to produce dense features that are similar in size to the input image. The details of the simplified architecture are tabulated in Table 1.

## 3. Experimental Setup

For the experiments, T1-weighted structural brain MRI data were used that are available on OASIS (open access series of imaging studies). OASIS is an open access website [12], created by the Alzheimer's Disease Research Center at Washington University. The dataset consists mainly of brain MRI images from Alzheimer's disease patients aged 18 to 96 and normal human brain MRI images for comparative study. All experiments were conducted using Matlab R2017b on an i3 4160, 4 GB RAM windows desktop. To reduce computation time, the neural network was trained by a single GeForce GTX 1050 Ti GPU using parallel computing.

### 3.1. Image Extraction and Preprocessing

The dataset consisted of several types of MRI scans with raw, processed, and segmented 3D raw files or analyze format file (.img, .hdr). Cross-sectional averaged and coregistered scan images were used that were obtained in the native acquisition space resampled to 1 mm isotropic voxels [12] from 50 subjects (cross-sectional MRI brain scans of dimensions 208 × 176 × 160). The MRIcon software package was used to extract slices from each mid cross-sectional MRI to generate images of size 208 × 176 pixels, each representing a single MRI scan.

**Figure 3:**
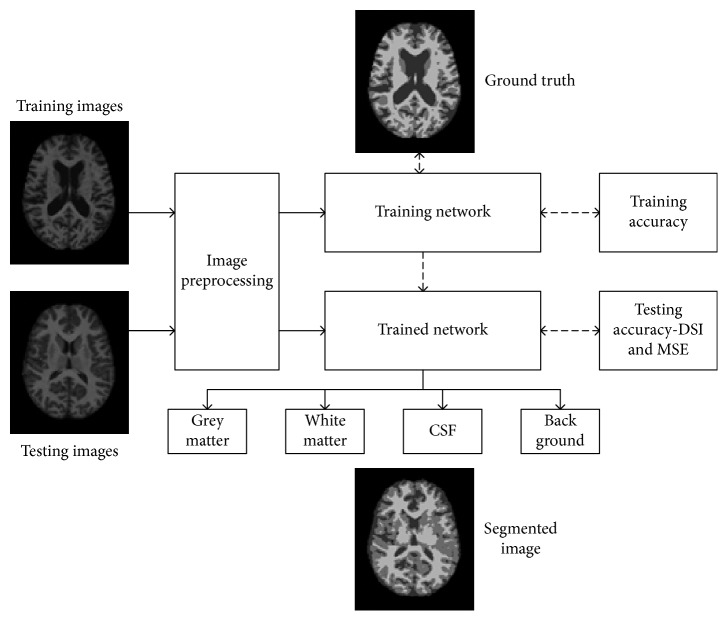
Representation of the proposed method, the training and testing image is separated, so that training images after preprocessing feeds into CNN network along with its respective ground truth, after reaching to convergence, the training is stopped and the network is now called trained. This trained network is used to test other test image separately to get segmented image, which is compared with its ground truth itself for performance analysis.

Two disc images were selected from ID OAS1_0001_MR1 to OAS1_0080_MR1, consisting of 76 images originally. The skull stripped image was used as training image, and the segmented images (the image is already segmented into four parts) of each training image were used as training labels or ground truth. Later, the trained network was used to segment the test MRI images, and the result was compared with the ground truth segmentation. Regarding the training environment, “Stochastic Gradient Descent with Momentum” was selected as the training optimization algorithm, with an initial learning rate of 0.001. To facilitate smooth training, the training was carried out in minibatches of 8 files per epoch, with data augmentation carried out at a random reflection in *X*-axis and rotation of ±10 degrees from the original position of each image. The predesigned SegNet layers created a training network, which was to undergo a stepwise feature extraction process on each CNN layer. Additionally, the classification of pixels was facilitated by classweight−classname pairs and a cross-entropy loss function. The SegNet layer acted as the training framework, whereas the pixel classification layer acted as classification output. The overall workflow of proposed method is illustrated in Figure 3.

### 3.2. Training and Testing Accuracy

**Figure 4:**
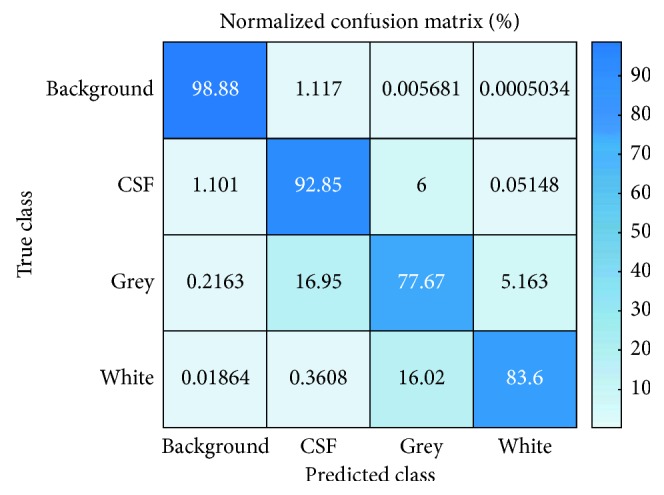
Confusion matrix of the experiment. The diagonal represents the accuracy of predicted class versus true class.

Seventy-six images were selected from ID OAS1_0001_MR1 to OAS1_0080_MR1 for training (including the augmented images) excluding four missing MRI and six images from ID OAS1_0081_MR1 to OAS1_0087_MR1 for testing excluding OAS1_0082_MR1. The overall training accuracy was 91.47 with mean global accuracy 0.91, mean accuracy 0.88248, mean IoU 0.88248, and WeightedIoU 0.84. The clusterwise accuracy, IoU, and MeanBFScore are tabulated in Table 2. The intersection over union (IoU) for the best predicted image was approximately 0.8477, whereas IoU for the worst predicted image was approximately 0.625. The confusion matrix obtained from the classification is shown in Figure 4. The obtained result shows, out of total training image, the network could correctly classify around 99% of background pixel, 93% of CSF pixel, 78% of GM, and 83.6% of WM which indicates the network is trained and ready to perform segmentation in other testing image.

### 3.3. Dice and Jaccard Similarity Index

To assess the performance of the method, the Dice similarity index, the Jaccard coefficient, and the mean squared error (MSE) of each tested image were calculated with reference to the ground truth image available in the same database. For comparison, each image was converted into a label image as that of ground truth. From the experiment, it can be clearly seen that results of high visual quality were obtained, with almost 80% Dice similarity index in each test image.

The Dice similarity coefficient of two sets *x* and *y* is defined as

(1) $$\begin{array}{c} \text{Dice}\left(x,y\right)=2\;\times \;\left|\text{intersection}\left(x,y\right)\right|/\left(\left|x\right|+\left|y\right|\right)\text{,} \end{array}$$

where |*x*| represents the cardinality of the set *x* and |*y*| represents the cardinality of the set *y*.

Similarly, the Jaccard similarity coefficient is defined as

(2) $$\begin{array}{c} \text{Jaccard}\left(x,y\right)=\left|\text{intersection}\left(x,y\right)\right|/\left|\text{union}\left(x,y\right)\right|. \end{array}$$

MSE is defined as

(3) $$\begin{array}{c} \text{MSE}=\frac{1}{M\times N}\overset{M}{\underset{i=1}{\sum }}\overset{N}{\underset{j=1}{\sum }}\left(I-I^{\prime }\right), \end{array}$$

where *I* and *I*′ stand for the pixel intensity value for the ground truth reference image of size *M∗N* and the simulated image pixel value of the same image size, that is, *M∗N*, respectively. Both the Dice similarity index and the Jaccard similarity index are important parameters for determining how closely the images *I* and *I*′ are related, and IoU is used for determining how closely they are spatially matched, with no wrong mapping. Similarly, MSE was calculated to authenticate the similarity index and the resemblance of the simulated result *I*′ to the ground truth *I* with minimum loss of information.

## 4. Results and Discussion

**Figure 5:**
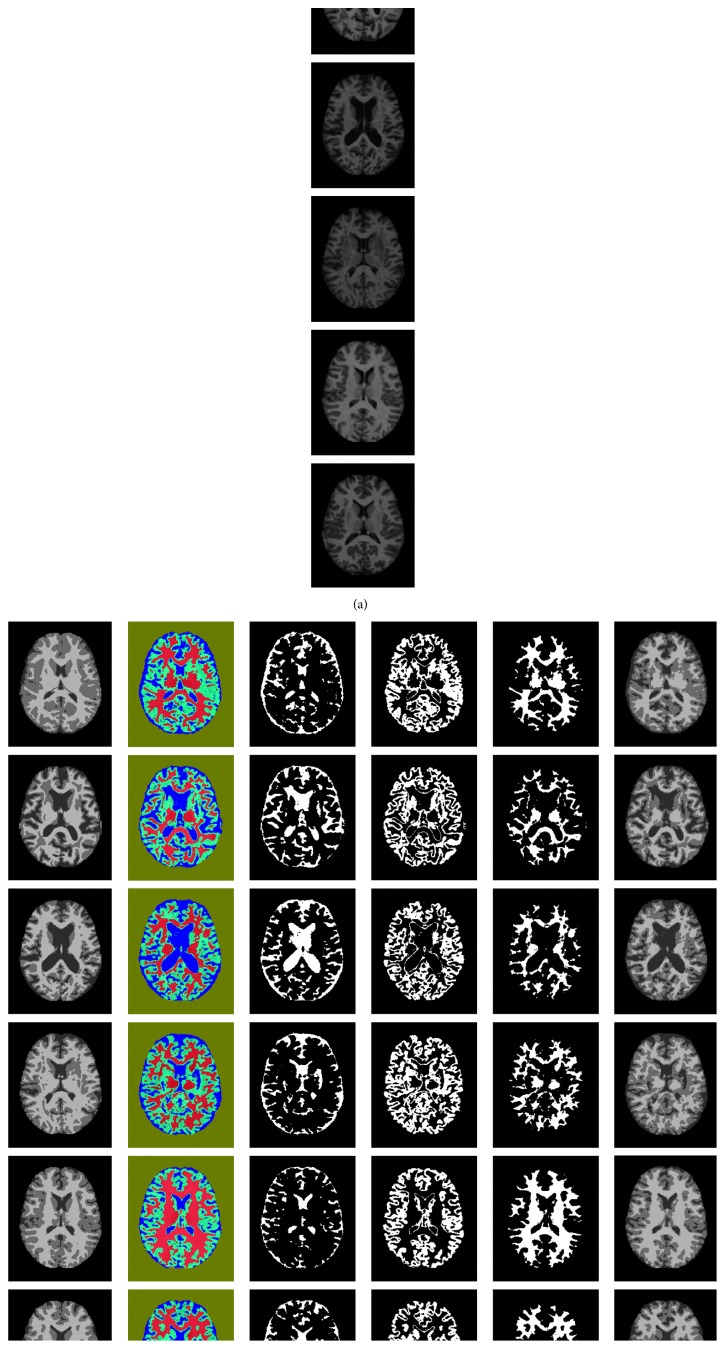
Original image and ground truth image presented along with other images, as a result of proposed segmentation: (a) original image, (b) ground truth, (c) segmented image by proposed method (color), (d) CSF part (binary image), (e) GM part (binary image), (f) WM part (binary image), and (g) segmented Image in gray (c).

The results obtained appear satisfactory and visually distinguishable. Figures 5(a)–5(g) show the results of the experiment. The first column (a) contains the original MRI images obtained from the OASIS database, which are cross-sectional T1 images; the next column (b) contains the ground truth or the segmented image of respective images in column (a). The third column (c) shows the main results, which are segmented using the proposed method, that is, segmentation based on pixel label. The remaining three columns (d), (e), and (f) show the extracted binary image as a classification result of (c). The segmented image (c) is represented by gray level intensity in (g), which is compared with the ground truth to evaluate the Dice similarity coefficient (DSC) of each class, namely, WM, GM, and CSF.  Table 3 presents the performance parameter for each image presented in row (a) of the original image in Figure 5.

### 4.1. Comparison with Other State-of-the-Art Methods

The computed mean DSC was approximately 80% (highest 84% and lowest 71%) among 6 test images. To compare this result, similar previous approaches for brain image segmentation are presented in Table 4. Zhang et al. [12] used a patchwise CNN for private data of 10 healthy infants, and Nie et al. [13] used semantic approach for the same type of data. Our approach was superior to those by de Brebisson et al. [14] and Moeskops et al. [15] in terms of DSC, but the dataset used here is OASIS mid cross-sectional T1 MRI 2D images instead of MICCAI 2012 Atlas.

## 5. Conclusions

In conclusion, we have successfully applied deep learning technique for image segmentation with convincing results. Specifically, we are able to segment closely related brain MRI images on pixel-label basis using encoder-decoder network of SegNet layer, which is generally used in semantic segmentation of outdoor scene. This suggests us that, with certain modification and simplified architecture, deep neural network can be effective in medical MRI image segmentation as like natural outdoor images.

## Figures and Tables

**Table 1:** Simplified SegNet network.

S. No.	Layer name	Type	Description
1	“Image input”	Image	208 × 1761 images with “zero center” normalization
2	“encoder1_conv1”	Convolution	64 3 × 3 × 1 convolutions with stride [1 1] and padding [1 1 1 1]
3	“encoder1_bn_1”	Batch normalization	Batch normalization with 64 channels
4	“encoder1_relu_1”	ReLU	ReLU
5	“encoder1_conv2”	Convolution	64 3 × 3 × 64 convolutions with stride [1 1] and padding [1 1 1 1]
6	“encoder1_bn_2”	Batch normalization	Batch normalization with 64 channels
7	“encoder1_relu_2”	ReLU	ReLU
8	“encoder1_maxpool”	Max pooling	2 × 2 max pooling with stride [2 2] and padding [0 0 0 0]
9	“encoder2_conv1”	Convolution	64 3 × 3 × 64 convolutions with stride [1 1] and padding [1 1 1 1]
10	“encoder2_bn_1”	Batch normalization	Batch normalization with 64 channels
11	“encoder2_relu_1”	ReLU	ReLU
12	“encoder2_conv2”	Convolution	64 3× 3 × 64 convolutions with stride [1 1] and padding [1 1 1 1]
13	“encoder2_bn_2”	Batch normalization	Batch normalization with 64 channels
14	“encoder2_relu_2”	ReLU	ReLU
15	“encoder2_maxpool”	Max pooling	2 × 2 max pooling with stride [2 2] and padding [0 0 0 0]
16	“decoder2_unpool”	Max unpooling	Max unpooling
17	“decoder2_conv2”	Convolution	64 3× 3 × 64 convolutions with stride [1 1] and padding [1 1 1 1]
18	“decoder2_bn_2”	Batch normalization	Batch normalization with 64 channels
19	“decoder2_relu_2”	ReLU	ReLU
20	“decoder2_conv1”	Convolution	64 3 × 3 × 64 convolutions with stride [1 1] and padding [1 1 1 1]
21	“decoder2_bn_1”	Batch normalization	Batch normalization with 64 channels
22	“decoder2_relu_1”	ReLU	ReLU
23	“decoder1_unpool”	Max unpooling	Max unpooling
24	“decoder1_conv2”	Convolution	64 3 × 3 × 64 convolutions with stride [1 1] and padding [1 1 1 1]
25	“decoder1_bn_2”	Batch normalization	Batch normalization with 64 channels
26	“decoder1_relu_2”	ReLU	ReLU
27	“decoder1_conv1”	Convolution	4 3 × 3 × 64 convolutions with stride [1 1] and padding [1 1 1 1]
28	“decoder1_bn_1”	Batch normalization	Batch normalization with 4 channels
29	“decoder1_relu_1”	ReLU	ReLU
30	“Softmax”	Softmax	Softmax
31	“Pixel_classify”	Pixel classification layer	Class weighted cross-entropy loss with “background,” “CSF,” “GM,” and “WM” classes

**Table 2:** Training accuracy, intersection over union (IoU), and MeanBFscore for each assigned class.

	Accuracy	IoU	MeanBFScore
Background	0.98877	0.9855	0.99456
CSF	0.92848	0.66983	0.8923
Gray	0.77666	0.66022	0.93897
White	0.83603	0.79073	0.90347

**Table 3:** Comparison of performance parameters for each result image (Figure 5(g)), with respective ground truth image (Figure 5(b)).

Test image ID	Parameter	CSF part	Gray part	White part	Mean value
OAS1_0081_MR1	Dice similarity	0.54	0.75	0.85	0.71
	Jaccard similarity	0.37	0.59	0.74	0.57
	Mean squared error	—	—	—	29.47

OAS1_0083_MR1	Dice similarity	0.84	0.75	0.79	0.80
	Jaccard similarity	0.73	0.60	0.66	0.66
	Mean squared error	—	—	—	19.32

OAS1_0084_MR1	Dice similarity	0.85	0.71	0.78	0.78
	Jaccard similarity	0.74	0.55	0.64	0.64
	Mean squared error	—	—	—	25.02

OAS1_0085_MR1	Dice similarity	0.72	0.67	0.73	0.71
	Jaccard similarity	0.56	0.51	0.57	0.55
	Mean squared error	—	—	—	32.52

OAS1_0086_MR1	Dice similarity	0.74	0.85	0.92	0.84
	Jaccard similarity	0.59	0.74	0.85	0.73
	Mean squared error	—	—	—	9.52

OAS1_0087_MR1	Dice similarity	0.64	0.75	0.85	0.74
	Jaccard similarity	0.47	0.60	0.74	0.60
	Mean squared error	—	—	—	27.58

**Table 4:** Comparison of deep learning approaches for brain structure segmentation.

Authors	CNN style	Dimension	Accuracy	Data
Zhang et al. [13]	Patchwise	2D	DSC 83.5% (CSF), 85.2% (GM), 86.4% (WM)	Private data (10 healthy infants)
Nie et al. [14]	Semantic-pixelwise	2D	DSC 85.5% (CSF), 87.3% (GM), 88.7% (WM)	Private data (10 healthy infants)
de Brebisson et al. [15]	Patchwise	2D/3D	Overall DSC 72.5% ∓ 16.3%	MICCAI 2012-multi-atlas labeling
Moeskops et al. [16]	Patchwise	2D/3D	Overall DSC 73.53%	MICCAI 2012-multi-atlas labeling
Proposed method	Pixel-label semantic (SegNet CNN)	2D	DSC 72.2% (CSF), 74.6% (GM), 81.9% (WM)	OASIS cross-sectional MRI

## Data Availability

The Open Access Series of Imaging Studies (OASIS) data were acquired through grants: P50 AG05681, P01 AG03991, R01 AG021910, P20 MH071616, and U24 RR021382. The MRI analyze format file (.img, and .hdr) data used in preparation of this article are publicly available on the OASIS database (http://www.oasis-brains.org/#data).
